# Regulation of myoblast differentiation by metabolic perturbations induced by metformin

**DOI:** 10.1371/journal.pone.0182475

**Published:** 2017-08-31

**Authors:** Theodora Pavlidou, Marco Rosina, Claudia Fuoco, Giulia Gerini, Cesare Gargioli, Luisa Castagnoli, Gianni Cesareni

**Affiliations:** 1 Laboratory of Molecular Genetics, Department of Biology, Tor Vergata University, Rome, Italy; 2 IRCCS, Fondazione Santa Lucia, Rome, Italy; Merck & Co., UNITED STATES

## Abstract

The metabolic perturbation caused by calorie restriction enhances muscle repair by playing a critical role in regulating satellite cell availability and activity in the muscles of young and old mice. To clarify the underlying mechanisms we asked whether myoblast replication and differentiation are affected by metformin, a calorie restriction-mimicking drug. C2C12, a mouse myoblast cell line, readily differentiate *in vitro* and fuse to form myotubes. However, when incubated with metformin, C2C12 slow their replication and do not differentiate. Interestingly, lower doses of metformin promote myogenic differentiation. We observe that metformin treatment modulates the expression of cyclins and cyclin inhibitors thereby inducing a cell cycle perturbation that causes a delay in the G2/M transition. The effect of metformin treatment is reversible since after drug withdrawal, myoblasts can re-enter the cell cycle and/or differentiate, depending on culture conditions. Myoblasts cultured under metformin treatment fail to up-regulate MyoD and p21cip1, a key step in cell cycle exit and terminal differentiation. Although the details of the molecular mechanisms underlying the effect of the drug on myoblasts still need to be clarified, we propose that metformin negatively affects myogenic differentiation by inhibiting irreversible exit from the cell cycle through reduction of MyoD and p21cip1 levels.

## Introduction

Skeletal muscle, upon damage or stress, activates a complex cross talk between heterogeneous populations of mononuclear cells that eventually triggers the activation of a specialized population of myogenic progenitors, the satellite cells (SC) [1]. The satellite cells are mitotically quiescent and upon activation by regenerative signals they can divide asymmetrically to reconstitute the pool of quiescent satellite stem cells, on one hand and, on the other hand, to give rise to myogenic cells (myoblasts) which proliferate, differentiate and fuse to pre-existing myofibers or form new myofibers [1]. C2C12 is a primary line of murine myoblasts that is often used as a myoblast model since it mimics *in vitro* the *in vivo* differentiation process. After a few days of growth most C2C12 cells pause in the G1 phase, exit permanently the cell cycle and eventually differentiate and fuse to form multinucleated myotubes [2]. Differentiating C2C12 cell cultures contain a sub-population of non-cycling, undifferentiated cells, called “reserve cells”, which share many characteristics of muscle satellite cells. Reserve cells are arrested in G0 but they can re-enter and progress through the cycle and eventually form both differentiated myotubes and mononucleated myoblasts [3]. The defining characteristic that distinguishes the quiescent satellite cells and the reserve cells from the cell cycle arrested myoblasts is reversibility.

Myogenic differentiation is a highly orchestrated process that depends on a family of myogenic regulatory factors (MRFs) such as MyoD, myogenin, Mrf4 and Myf5 [4]. Proliferating myoblasts express MyoD and Myf5 before the onset of myogenic differentiation [5]. Moreover, skeletal muscle regeneration and myogenic differentiation *in vitro* are tightly coupled to cell cycle regulation. The up-regulation of the cyclin-dependent kinase inhibitor p21cip1 (p21) and the dephosphorylation of retinoblastoma protein pRB (Rb1) are critical regulatory events that promote the establishment of the post-mitotic state during myogenic differentiation [6]. However, over recent years a different role for p21 has emerged, as it seems to be responsible for the establishment of the differentiation program without being necessary for the permanent proliferation arrest [7]. Specifically, p21cip1 (CDKN1A) and p57kip2 (CDKN1C) have been shown to redundantly control myogenic differentiation [8]. The transcriptional up-regulation of p21cip1 during myogenic differentiation is induced by MyoD [9].

Cerletti and colleagues reported that short-term calorie restriction enhances the number and the myogenic potential of Pax-7 expressing cells in the muscles of young and old mice [10]. They also observed an associated increase in mitochondrial abundance and an enhancement of transplant efficiency of SC cells of mice on a low-calorie diet. However, the role of different calorie restriction mimics on skeletal muscle regeneration is still controversial. Lately, pharmacological AMP-dependent kinase (Ampk) activation has been proposed as a potential therapeutic approach in Duchenne muscular dystrophy as it favors the slow, oxidative muscle phenotype which is more resistant to dystrophin defects [11]. Intraperitoneal injections of AICAR (an Ampk agonist) improved muscle integrity and reduced muscle degeneration in mdx mice [12]. Our group demonstrated that metformin treatment limits cardiotoxin damage by protecting myotubes from necrosis, without significantly influencing muscle regeneration [13]. Building on these observations, we asked whether metformin, a calorie restriction-mimicking drug, could affect the proliferation and differentiation of myoblasts *in vitro*.

Metformin (1,1-dimethylbiguanide), a biguanide derivate, is the most widely used treatment for hyperglycemia in individuals with Type II Diabetes [14]. The main action of metformin is to reduce hepatic glucose production, mainly by inhibiting gluconeogenesis [15]. It is still ambiguous which is the exact mechanism by which metformin reduces hepatic glucose production. However, hepatocyte mitochondria seem to be the primary target of metformin, where it inhibits respiratory complex I chain oxidation [16]. This results in accumulation of AMP and activation of Ampk [17]. AMP-activated protein kinase (Ampk) is a serine/threonine kinase which is modulated by changes in the levels of AMP and/or ATP and, as such, works as a sensor of cellular energy status and metabolic stress [18]. It has been also reported that metformin may have beneficial effects on the prevention and treatment of cancer as it inhibits the proliferation and cyclin D1 expression of diverse cultured cancer cells [19]. Given the widespread use of metformin as an antidiabetic drug, understanding secondary effects on different tissues is of primary importance.

Here we propose that, *in vitro*, metformin impairs the growth and the skeletal muscle differentiation of C2C12 myoblasts by delaying them in the G2/M phase of the cell cycle, thereby inhibiting their permanent exit from the cell cycle and down-regulating p21cip1 expression. Interestingly, the differentiation block of myoblast treated with metformin is reversible, thereby mimicking the phenotype of “reserve cells”.

## Materials and methods

### Cell culture

C2C12 myoblasts were purchased from ATCC (American Type Culture Collection, Bethesda, MD, USA) (CRL-1772). The mesoangioblast cell line was kindly provided by Giulio Cossu’s laboratory [20]. The cells were seeded on Falcon dishes at 37°C with 5% CO_2_ in growth medium (GM, Dulbecco modified Eagle medium, DMEM), supplemented with 10% heat-inactivated fetal bovine serum, (100 U/100 g/ml) penicillin-streptomycin, 1mM sodium pyruvate and 10mM HEPES. For differentiation experiments, growth medium was replaced by differentiation medium (DM, DMEM with 2% horse serum) when cells reached 80% confluence. The following 3 days, the cells were treated with or without metformin at the final concentration of 0.1, 2 and 10mM. Metformin was added fresh to the medium every 24h.

### Proliferation assay

C2C12 myoblasts were seeded at 4158 cells/cm^2^ in growth medium (GM) on 6-well plate. After 24h, cells were treated with 100 μM, 500 μM, 2 mM, 5 mM and 10 mM metformin for 4 days in GM. Metformin was added to the medium every 24h. At each time-point cells were fixed with 2% paraformaldeyde solution in PBS 1X for 10 minutes at room temperature (RT) and stored at 4°C in PBS 1X. At the end of the experiment all samples were stained with 2 μg/ml Hoechst 33342 (ThermoFisher Scientific) in Triton X-100 0.1% (v/v) in PBS 1X for 5 minutes at RT and images acquired with a Leica DM6000B (Leica Microsystems) automated fluorescence microscope. 25 fields/well were automatically acquired using a 5x5 matrix covering the whole surface of the specimen. Nuclei were counted with Cell Profiler software. The experiment was performed at least three times.

### Immunofluorescence

C2C12 myoblasts and myotubes were fixed with 2% paraformaldeyde (PFA) for 15min and permeabilized in 0.1% Triton X-100 for 5min. Cells were then blocked with 1% PBS 10% Serum 0,1% TritonX-100 for 1h at room temperature (RT). Incubation with the primary antibody was performed for 1h at RT, cells were washed three times and incubated with the secondary antibody for 30min at RT. The antibodies used were the following: rabbit anti-Ki67 (1:150, NovusBiologicals NB110-89717), rabbit anti-MyoD (1:20, Santa Cruz sc-760), mouse anti-Myosin Heavy Chain, MHC (1:2 MF20, DSHB), anti-rabbit secondary antibody Alexa Fluor 555 conjugated (1:100, Life technologies A-21428) and anti-mouse secondary antibody Alexa Fluor 488 conjugated (1:100, Life technologies A-11001). The samples were washed three times and nuclei were counterstained with Hoechst 33258 (1 mg/ml, 5min at RT). Images were acquired with LEICA fluorescent microscope (DMI6000B).

### Immunoblotting

C2C12 cells were washed in the culture dish with ice-cold PBS and homogenized in RIPA lysis buffer (150mM NaCl, 50mM Tris-HCl, 1% Nonidet P-40, 0.25% sodium deoxycholate) supplemented with 1mM pervanadate, 1mM NaF, protease inhibitor cocktail 200X (Sigma), phosphatase inhibitor cocktail I and II 100X (Sigma). Samples were incubated in ice for 30min with the lysis buffer and cell debris were separated by centrifugation at 14,000 rpm for 30min at 4°C. The supernatant was collected and stored at -80°C. Protein concentrations were determined by Bradford colorimetric assay (Bio-Rad). Total protein extracts (30μg) were then separated by SDS-PAGE. Gels were transferred to membranes, saturated with blocking solution (5% milk and 0.1% Tween-20 in PBS) and incubated with primary antibodies overnight at 4°C. The antibodies used were as follows: rabbit anti-MyoD (1:500, Santa Cruz sc-760), mouse anti-myogenin (1:500, e-Bioscience 14–5643), mouse anti-MHC (1:5 MF20, DSHB), rabbit anti-Myf5 (1:500, Santa Cruz sc-302), rabbit anti-phospho Ampk (Thr172) antibody (1:1000, Cell Signaling 2535), rabbit anti-Ampk (1:1000, Cell Signaling 2603), rabbit anti-phospho P70S6K (Thr421/Ser424) (1:1000, Cell Signaling 9204), rabbit anti-P70S6K (1:1000, Cell Signaling 9202), rabbit anti-phospho RPS6 (Ser240/244) antibody (1:1000, Cell Signaling 2215), rabbit anti-RPS6 (1:1000, Cell Signaling 2217), mouse anti-p27kip1 (1:1000, BD bioscience 610241), mouse anti-p21cip1 (1:500, Santa Cruz sc-6246), rabbit anti-cyclin D3 (1:500, Santa Cruz sc-182), rabbit anti-cyclin B (1:1000, Cell Signaling 4138), rabbit anti-Cdc2 (1:1000 Santa Cruz sc-954), rabbit anti-p130 (1:500, Santa Cruz sc-317), rabbit anti-phospho p38 (1:1000, Cell Signaling 9211), rabbit anti-p38 (1:1000, Cell Signaling 9212), rabbit anti-cleaved caspase 3 (1:1000, Cell Signaling 9664), rabbit anti-cleaved caspase 3 (1:1000, Cell Signaling 8438), mouse anti-vinculin (1:2000, Chemicon MAB1624) and rabbit anti-tubulin antibody (1:500, Santa Cruz sc-9104). The membranes were then washed three times and incubated with anti-mouse or anti-rabbit secondary antibody conjugated with HRP (1:2500, Jackson ImmunoResearch) for 1h at RT. The blots were further washed three times and visualized with an enhanced chemiluminescent immunoblotting detection system. Densitometric analysis was performed using ImageQuant. Phosphorylated and total proteins were normalized with tubulin or vinculin. Finally the ratio between phosphorylated and total protein was determined.

### Apoptosis detection

*In situ* cell death detection kit, TMR red (Roche, Cat. No. 12 156 792 910) was used to quantitate the apoptotic DNA strand breaks (TUNEL technology) in control and metformin treated cells. After 3 days of treatment with metformin, cells were fixed in 4% PFA for 10min at RT and permeabilzed with 0,1% Triton X-100 in 0,1% sodium citrate for 2min on ice. Cells were further washed twice with PBS and incubated with freshly prepared TUNEL reaction mixture (90% Label Solution, 10% terminal deoxynucleotidyl transferase (TdT) enzyme solution) in a humidified atmosphere, for 60min, at 37°C in the dark. As positive control, were used cells that were previously incubated with 3U/μl DNAse I for 10min at RT. As negative control, were used cells incubated only with Label Solution, without the addition of TdT enzyme. Images were acquired with LEICA fluorescent microscope (DMI6000B).

### Cell cycle analysis

Myoblasts were trypsinized, washed three times with PBS and fixed in methanol/acetone (4:1) for 30min at 4°C. The cells were further washed two times with PBS and incubated with RNAse (100μg/ml) for 20min at RT. Finally they were incubated with 10% propidium iodide and the DNA content was measured by propidium iodide intensity by using a BD FACScalibur flow cytometer. The cell cycle phases were analyzed by the FlowJo software.

### Statistical analysis

All the data presented are mean values ± SEM of at least three independent experiments. Student’s t-test was used to determine significant differences between means in all experiments. The differences were considered significant at *p <0.05.

## Results

### Metformin blocks C2C12 growth without inducing apoptosis

**Fig 1:**
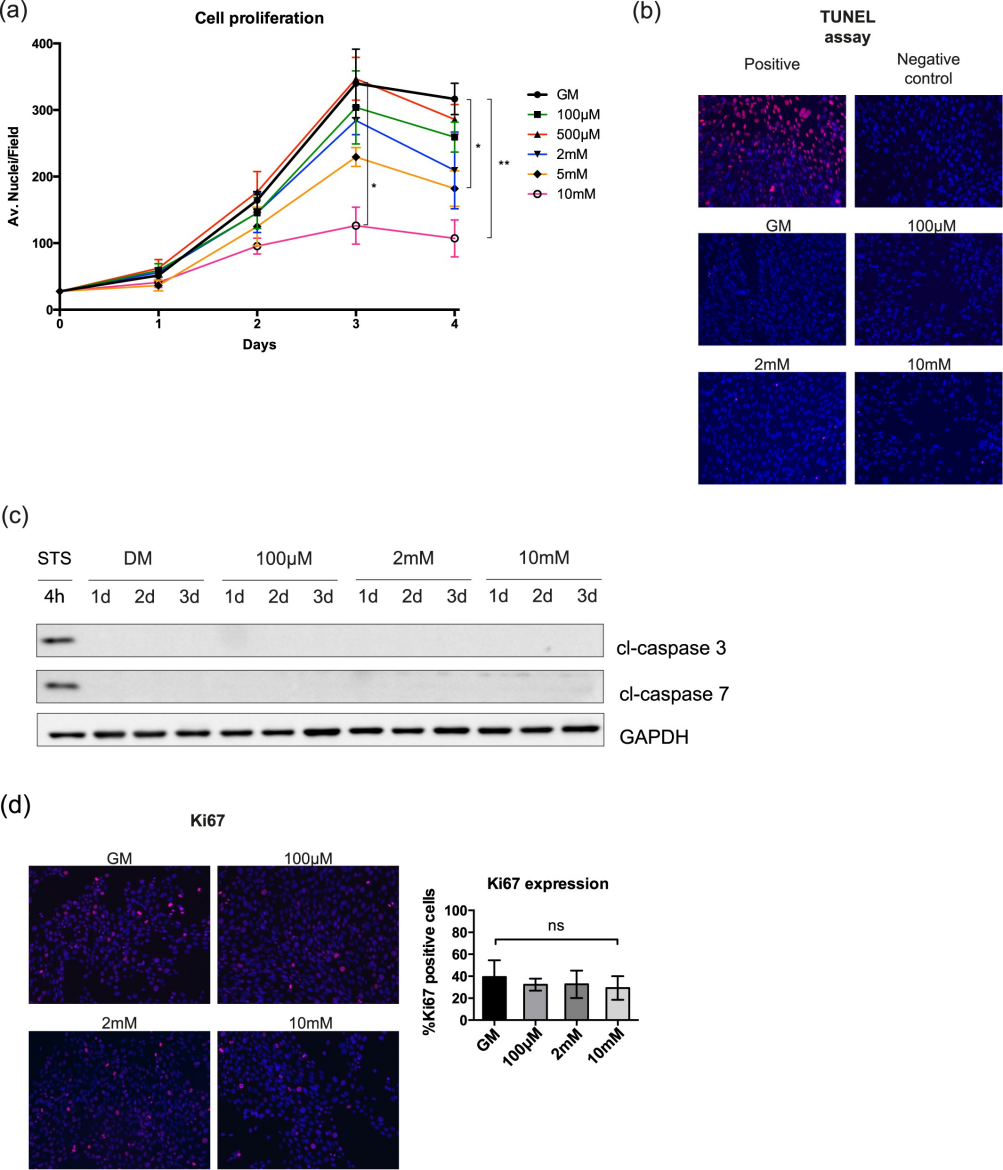
High doses of metformin inhibit the proliferation of C2C12 cells without inducing apoptosis. (a) C2C12 cells were treated with different doses of metformin in growth medium (GM) and the total number of cells was counted after 1, 2, 3 and 4 days of treatment by immunofluorescence microsopy. The initial number of plated cells was the same in each growth condition. Statistical significance was evaluated by the Student’s t-test (*p<0.05) (b) TUNEL assay. C2C12 cells were treated with 100μM, 2mM and 10mM metformin for 48h. As positive control for the TUNEL assay C2C12 myoblasts were incubated with DNΑse I before staining and, as negative control, cells were stained with the label solution without the addition of the reaction enzyme terminal deoxynucleotidyl transferase (TdT). (c) Total protein extracts of C2C12 myoblasts treated with 100μM, 2mM and 10mM were analyzed by SDS-PAGE for the expression of the apoptotic markers cl-caspase 3 and cl-caspase 7. For the induction of apoptosis in the positive control was used staurosporine 1μM for 4 hours. GAPDH is used as loading control (d) Proliferating C2C12 myoblasts, were plated at the same initial number (4*10^4^ in GM in 9,5 cm^2^ area wells), incubated with 100μM, 2mM and 10mM metformin for 48h. The percentage of cells expressing Ki67 was measured by Cell Profiler cell image analysis software. Statistical significance was evaluated by the Student’s t-test (*p<0.05).

Metformin has an anti-proliferative effect on tumor cells. As myoblast differentiation is dependent on irreversible exit from the cell cycle, we examined the proliferation of C2C12 myoblasts cultured in growth medium (GM) supplemented with different concentrations of metformin (100μM, 500μM, 2mM, 5mM and 10mM). The number of dead cells was monitored by the Trypan blue viability assay. No significant difference in the number of dead cells was observed even after treatment with high doses of metformin (S1 Fig). As observed by nuclear staining and immunofluorescence analysis, cells treated with 5mM and 10mM metformin are characterized by a statistically significant reduction in proliferation (Fig 1A). No positive signal for apoptosis was detected by the TUNEL assay for the whole range of tested drug concentrations (Fig 1B). This result was further confirmed by western blot analysis of the protein levels of cleaved caspase 3 and cleaved capase 7 (Fig 1C). Thus, most C2C12 cells in the presence of metformin either exit the cell cycle or are blocked/delayed in a phase of the cell cycle. We also monitored the expression of Ki67, a nuclear antigen that has a role in the regulation of higher-order chromatin structure and is expressed throughout the cell cycle of proliferating cells while it is not observed in resting cells [21]. Proliferating C2C12 myoblasts were treated with 100μM, 2mM and 10mM metformin for 48h and the expression of Ki67 was detected by immunofluorescence microscopy. As shown in Fig 1D the percentage of C2C12 cells positive for Ki67 is not significantly different in the treated samples when compared to the control. This suggests that the metformin treated C2C12 do not exit the cell cycle.

### Metformin impairs myogenic differentiation in C2C12 cells

**Fig 2:**
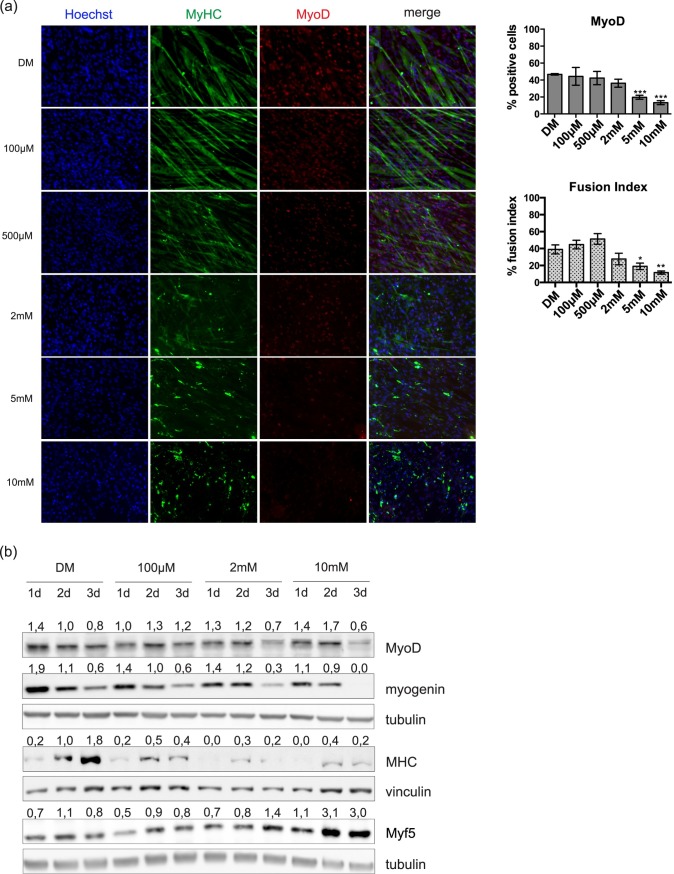
Metformin inhibits myogenic differentiation of C2C12 cells in a dose-dependent manner. (a) Immunofluorescence microscopy for the expression of the myogenic markers Myosin Heavy Chain (MHC) and MyoD. C2C12 induced to differentiate by serum deprivation in differentiation medium (DM) and treated for 3 days with different doses of metformin (100μM, 500μM, 2mM, 5mM and 10mM) were analyzed by immunofluorescence technique. The percentage of the cells positive for MyoD and the fusion index were determined using the Cell Profiler cell image analysis software. The fusion index was calculated as the % of the nuclei inside myotubes compared to the total number of nuclei. Only myotubes with at least three nuclei inside a continuous cell membrane were considered. Statistical significance was evaluated by the Student’s t-test (*p<0.05) (b) Western blot analysis of MyoD, myogenin, MHC and Myf5 in C2C12 myoblasts treated with different doses of metformin (100μM, 2mM and 10mM) for 3 days. Tubulin and vinculin are used as loading controls. Numbers above each band represent the quantitation of the densitometric analysis.

We next asked whether metformin also affects myogenic differentiation, as cell cycle exit and growth arrest are required for the activation of the myogenic gene cascade [22]. Proliferating myoblasts at ~ 80% confluence were induced to differentiate by incubation in differentiation medium (DM, 2% Horse serum in High Glucose DMEM), which has low concentrations of serum-derived mitogens. Given that C2C12 myoblasts start differentiating upon confluence, we plated the cells at high density in order to separate the effect of metformin on cell differentiation from the effect of the drug on cell proliferation. At the same time, the cells were treated with 100μM, 500μM, 2mM, 5mM or 10mM metformin for 3 days and the expression levels of myogenic factors were monitored. The expressions of the early marker MyoD as well as that of the late marker Myosin Heavy Chain (MHC) were detected by immunofluorescence microscopy. High doses of metformin (5mM and 10mM) inhibited myogenic differentiation, as demonstrated by the reduced fusion index and the significantly decreased expression of MyoD (Fig 2A). Interestingly, lower doses of the drug may effect myogenic differentiation as demonstrated by the trend toward increased fusion index in the sample treated with 500μM metformin, although this trend was not statistically significant. We also monitored the expression levels of myogenic markers by western blot analysis. Reduced expression of myogenin after 3 days and of MHC after 2 days are observed, following treatment with 2mM and 10mM metformin (Fig 2B). This is in accordance with the observation that myoblasts treated with these drug concentrations differentiate poorly. The expression levels of Myf5 by contrast are increased in the samples treated with the highest dose of metformin.

MyoD and Myf5 have opposite expression patterns in the different phases of the cell cycle in growing myoblasts and in primary satellite cells induced to differentiate [23]. Specifically, MyoD appears to undergo a bimodal pattern of expression after release from quiescence, with a peak in the middle of G1 and at the end of G2. On the contrary, the level of Myf5 is high in quiescent myoblasts, when MyoD is poorly expressed. MyoD reaches its highest levels in G1 while Myf5 is barely detectable in this phase to re-appear at the end of G1 and to stay constant over the S and G2 phases [24].

Our data suggest that metformin treated C2C12 cells do not differentiate and are characterized by low expression of MyoD and high expression of Myf5, an expression pattern that matches that of quiescent myoblasts and that of myoblasts in the S/G2 phase of the cell cycle.

### mTOR inactivation is induced by high doses of metformin

**Fig 3:**
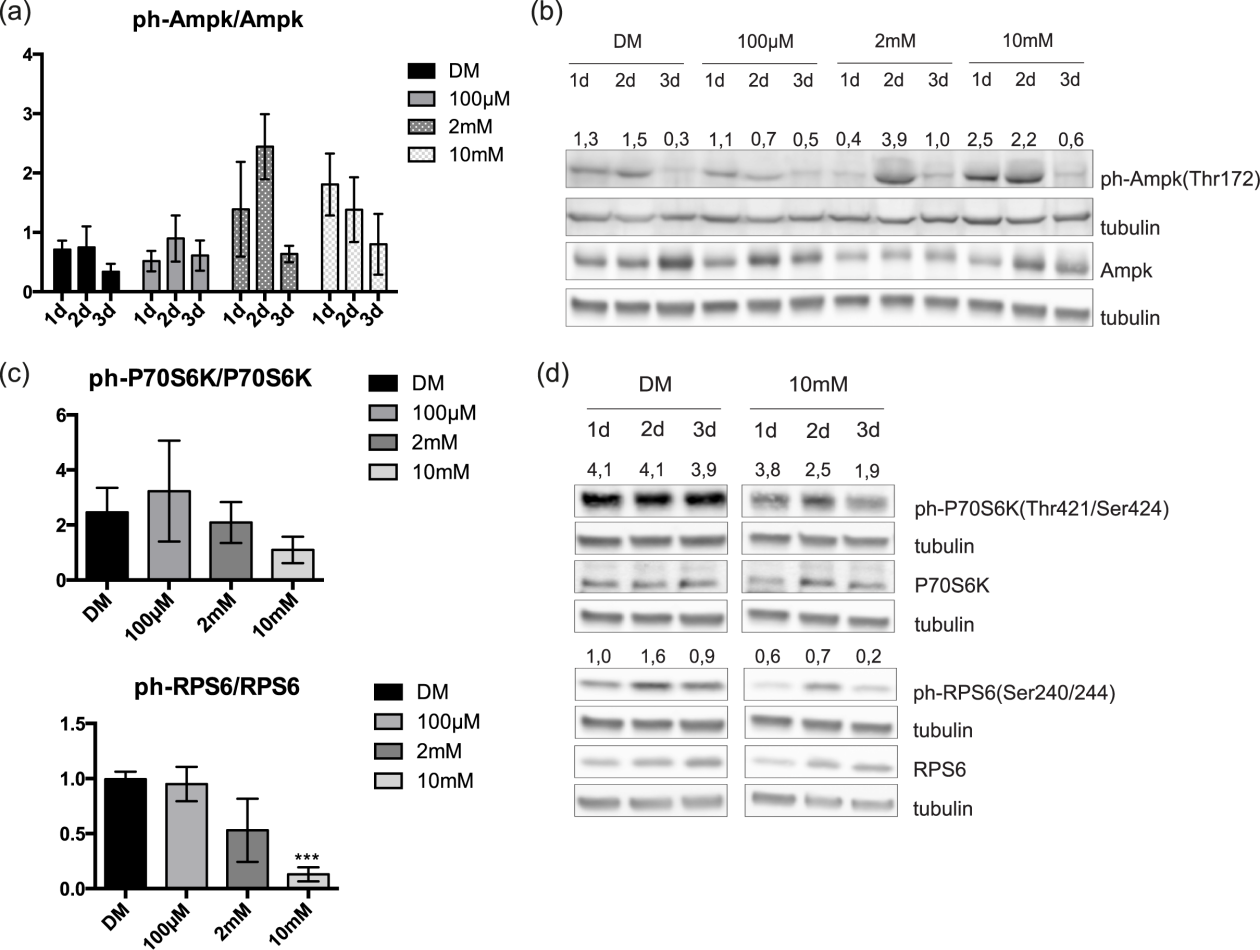
C2C12 response to metformin treatment. (a) Quantitation of ph-Ampk levels in three independent biological replicate experiments. Statistical significance was evaluated by the Student’s t-test (*p<0.05) (b) Western blot analysis of ph-Ampk and total Ampk in total protein lysates from metformin-treated and control C2C12 cells. C2C12 myoblasts, plated at the same density, when reached ~ 60% confluence were induced to differentiate by serum removal. Simultaneously, the cells were treated with 100μM, 2mM and 10mM metformin for 3 days. Metformin was refreshed every 24h and crude cell protein extracts were electrophoresed on SDS PAGE. Tubulin is used as loading control. Numbers above each lane represent the ratio of ph-Ampk/Ampk derived from the densitometric analysis (c) Quantitation of ph-P70S6K and ph-RPS6 levels after 3 days of metformin treatment in three independent biological experiments. Statistical significance was evaluated by the Student’s t-test (*p<0.05) (d) Western blot analysis of total P70S6K, RPS6 and their phosphorylation levels in crude protein extracts from control and treated with 10mM metformin C2C12 cells. Myoblasts induced to differentiate in low nutrient medium and treated with 10mM metformin for 3 days. Metformin was added fresh every 24h and total protein extracts were analyzed by SDS PAGE. Tubulin is used as a loading control. Numbers above each lane represent the ratio of ph-P70S6K/P70S6K and ph-RPS6/RPS6 derived from the densitometric analysis.

Ampk activation [17] and the ensuing inactivation of mTOR signaling [25] are hallmarks of cell response to metformin treatment. Therefore, we measured the phosphorylation of Ampk and of the downstream targets of mTOR, P70S6K and RPS6. Quantification of the western blot bands shows that the average values are consistent with the expected increment of Ampk phosphorylation on Thr172 (Fig 3A). Specifically, the levels of phosphorylated Ampk increase the second and the first day of treatment with 2mM and 10mM metformin, respectively (Fig 3B). This was accompanied by a decrease of P70S6K phosphorylation on Thr421/Ser424 and of RPS6 phosphorylation on Ser240/244 (Fig 3C), with most evident effects at 3 days of treatment with 10mM metformin (Fig 3D).

### Metformin delays C2C12 cells in G2/M phase

**Fig 4:**
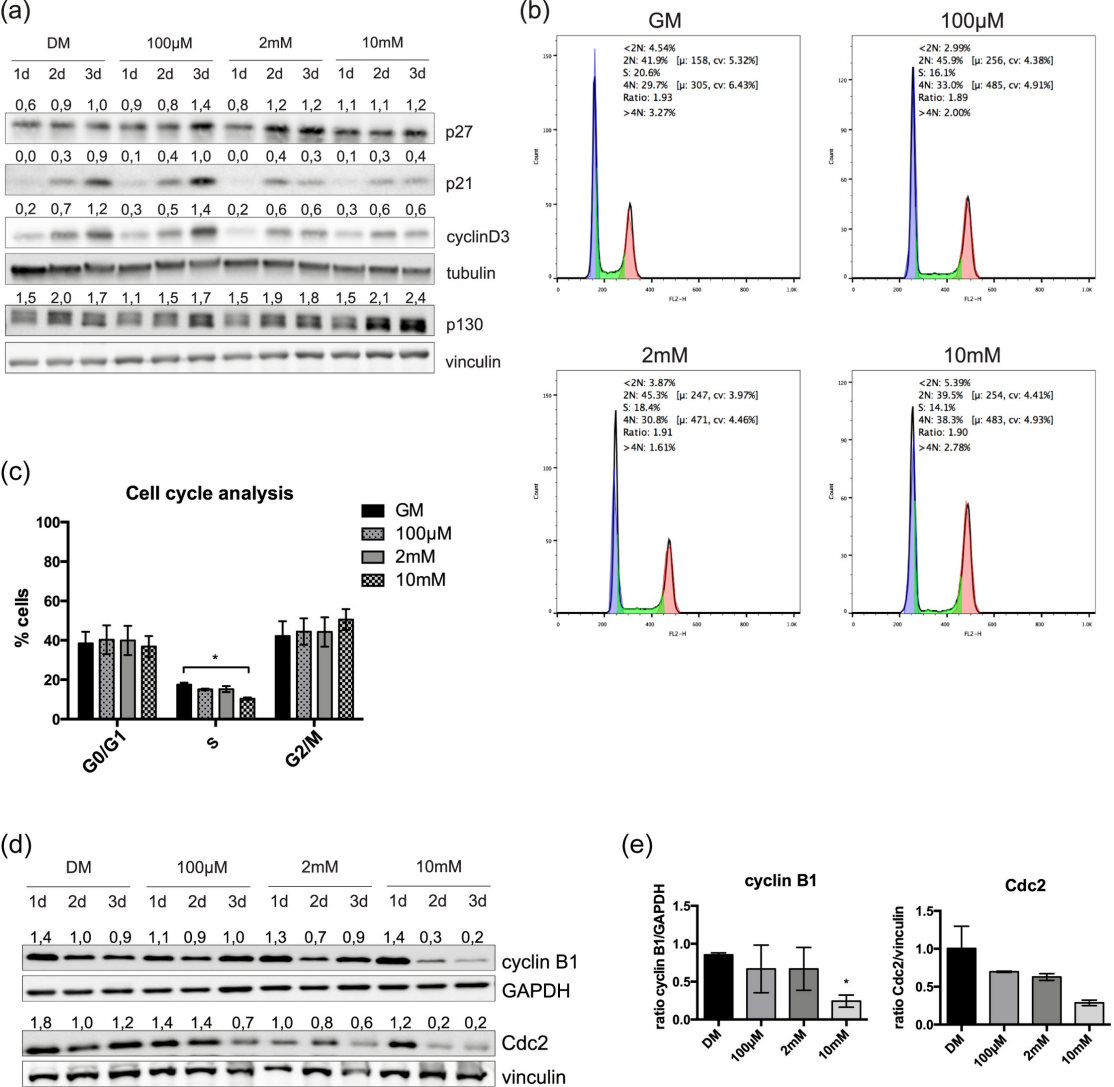
Metformin perturbs the expression of cyclins and CDKIs. (a) Western blot analysis of p27, p21, cyclin D3 and p130 in total protein lysate from metformin-treated and control C2C12 cells. When cells reached ~ 60% confluence, were induced to differentiate by serum deprivation and treated in differentiation medium with 100μM, 2mM and 10mM metformin for 3 days. Metformin was refreshed every 24h and crude cell protein extracts were analyzed by SDS PAGE. Tubulin and vinculin were used as loading controls. Numbers above each lane represent the densitometric analysis (b) Cell cycle analysis by DNA staining with propidium iodide. C2C12 cells were cultured for 3 days in GM and treated with 100μM, 2mM and 10mM metformin (c) Flow cytometry analysis of the three different phases of the cell cycle (G0/G1, S, G2/M) is represented by the histogram graph. Quantification of the different phases of the cell cycle was performed after three independent biological replicate experiments (d) Western blot analysis of the G2/M markers cyclin B and Cdc2 kinase in total protein lysate from metformin-treated and control C2C12 cells. Cells were induced to differentiate by serum deprivation and treated with 100μM, 2mM and 10mM metformin for 3 days. Metformin was added fresh every 24h and crude cell protein extracts were analyzed by SDS PAGE. GAPDH and vinculin were used as loading controls. Numbers above each lane represent the densitometric analysis (e) Quantitation of cyclin B and Cdc2 levels after 3 days of metformin treatment. Statistical significance was evaluated by the Student’s t-test (*p<0.05).

Since in the transition from proliferative myoblasts to post-mitotic myotubes the expression of many cyclins and CdKs is modulated [6], we investigated the expression of some of the cyclins and cyclin inhibitors that are involved in myogenic differentiation. The levels of cyclin D3, p21cip1 (p21) and p27kip1 (p27) were monitored by western blot. The expression level of the cyclin inhibitor p27 increased after metformin treatment (Fig 4A). On the other hand, the expression levels of p21 and cyclin D3 significantly decreased after 3 days of metformin treatment. Finally, investigating the expression of another cell cycle inhibitor, the retinoblastoma-related protein Rbl2 (p130), we noticed an increased p130 expression level upon treatment with 10mM metformin. The Rb-like protein p130 has been reported to be involved in the establishment of the muscle “reserve” cells by blocking cell cycle progression and differentiation [26]

Thus metformin treated myoblasts decrease their proliferation rate while they do not differentiate. We could hypothesize that these cells exit the growth cycle to rest in a reversible G0 state that mimics the quiescent state of the “reserve” cells. At the same time, however, these cells are stained with antibodies against the Ki67 antigen, which is not expressed in G0, suggesting that they are trapped/delayed in one phase of the cell cycle, other than G0. To obtain additional insights, we analyzed the cell distribution in the different phases of the cell cycle by flow cytometry. After 3 days of incubation in GM containing 10mM metformin, the fraction of cells in the G2/M phase is higher compared to the control, while the cells in the S phase are fewer (Fig 4B and 4C). To this end, we further monitored the effect of metformin on the expression of cyclin B and Cdc2 p34 kinase, two of the most essential regulators of the G2/M transition. Consistent with the previously observed delay in the G2/M phase of the cell cycle, we observed that 10mM metformin reduced the levels of cyclin B and Cdc2 p34 kinase (Fig 4D and 4E). Hence, metformin treatment delays cells in the G2/M phase thereby limiting cell cycle exit into an irreversible G0 phase.

### The effect of metformin is reversible

**Fig 5:**
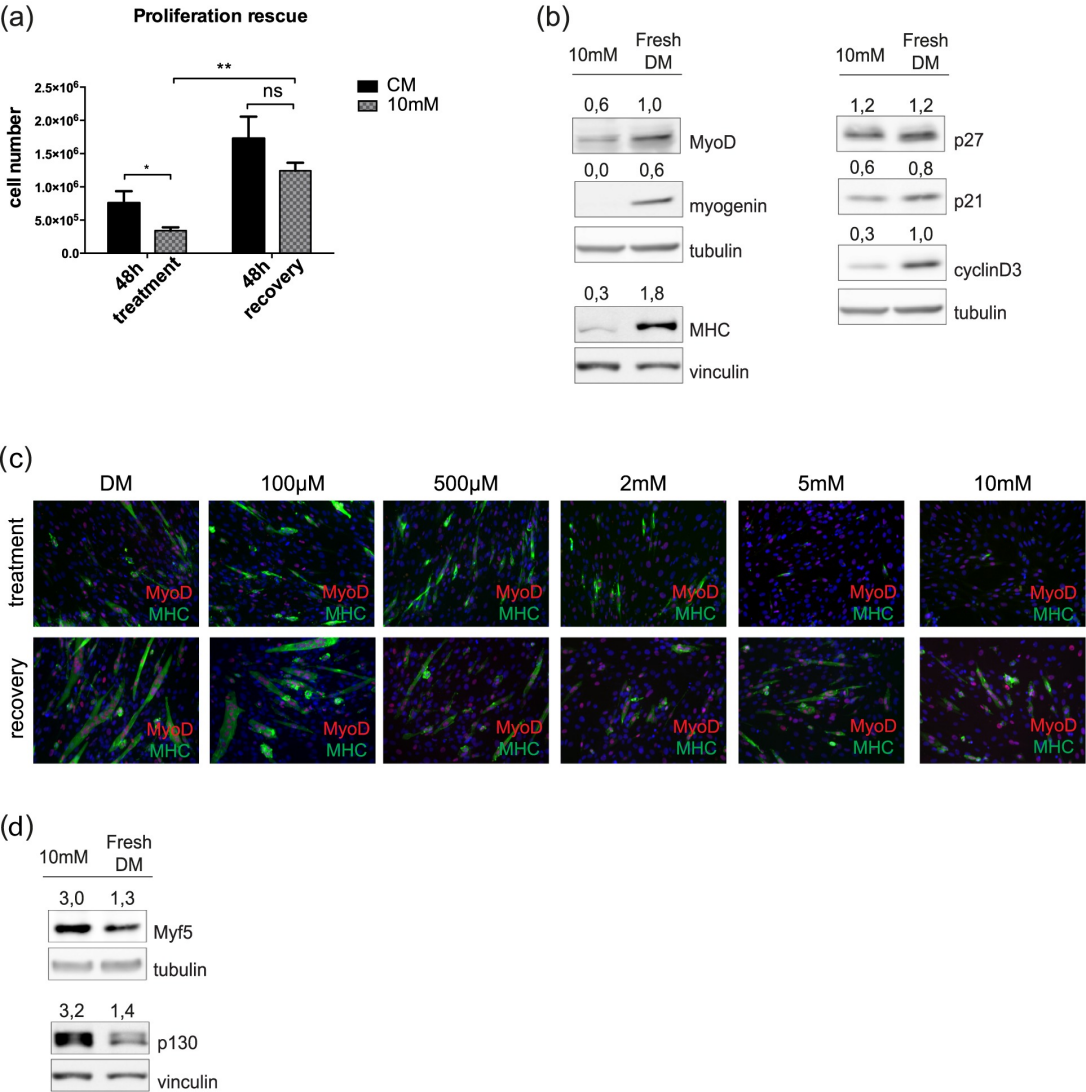
The metformin effect is reversible. (a) C2C12 myoblasts were treated with 10mM metformin in GM for 48h and subsequently let recover for additional 48h after replacing the medium containing the drug with fresh GM. After 48h of recovery, the total cell number was counted in the treated and control sample. Statistical significance was evaluated by the Student’s t-test (*p<0.05) (b) C2C12 induced to differentiate and treated with 10mM metformin for 72h let recover their differentiation potential for additional 48h in fresh DM. Protein extracts were analyzed by Western Blot. Tubulin and vinculin were used as loading controls. Numbers above each lane represent the densitometric analysis (c) The expression of the myogenic markers MyoD and MyHC and the formation of myotubes was analyzed by immunofluorescence microscopy. C2C12 myoblasts were treated with 100μM, 500μM, 2mM, 5mM and 10mM metformin in DM for 3 days and let recover for additional 72h (d) C2C12 myoblasts treated with 10mM metformin for 72h in differentiation medium, were subsequently cultured for additional 48h in fresh DM without metformin. The expression of Myf5 and p130 was measured by Western Blot. Tubulin and vinculin were used as loading controls. Numbers above each band represent the densitometric analysis.

The most defining characteristic that separates quiescence from other non proliferating cell states is reversibility [27]. On the contrary, terminal differentiation or senescence are characterized by an irreversible cell cycle arrest. In order to further clarify the cell state after metformin treatment, we investigated the reversibility of the metformin anti-proliferative effect. We treated C2C12 myoblasts with 10mM metformin in growth medium for 48h and subsequently we let them recover for additional 48h after replacing the medium containing the drug with fresh GM. The total cell number was determined and the number of dead cells was defined by the Trypan blue viability assay. No significant difference in the number of dead cells was observed. After replacing the culture medium, C2C12 cells recovered their proliferation potential and the total number of cells after recovery was not significantly different from that of the untreated sample (Fig 5A). In addition, we asked whether the inhibition of differentiation induced by metformin was a reversible process. After recovery for 48h in fresh DM, metformin treated cells rescued the expression of the myogenic markers MyoD, myogenin, MHC, cyclin D3 and cyclin inhibitor p21 while the level of p27 remained static (Fig 5B). In addition, immunofluorescence analysis revealed that C2C12 differentiate again into myotubes when grown in fresh DM for 48h (Fig 5C). Interestingly, the markers Myf5 and p130, which have been reported to characterize quiescence, were down-regulated upon cell recovery (Fig 5D). Taken together these results suggest that metformin induces a reversible impediment to cell proliferation that is distinct from the irreversible cell cycle exit leading to terminal differentiation.

### The differentiation of C2C12 cells, after metformin withdrawal, does not require phosphorylation of p38

**Fig 6:**
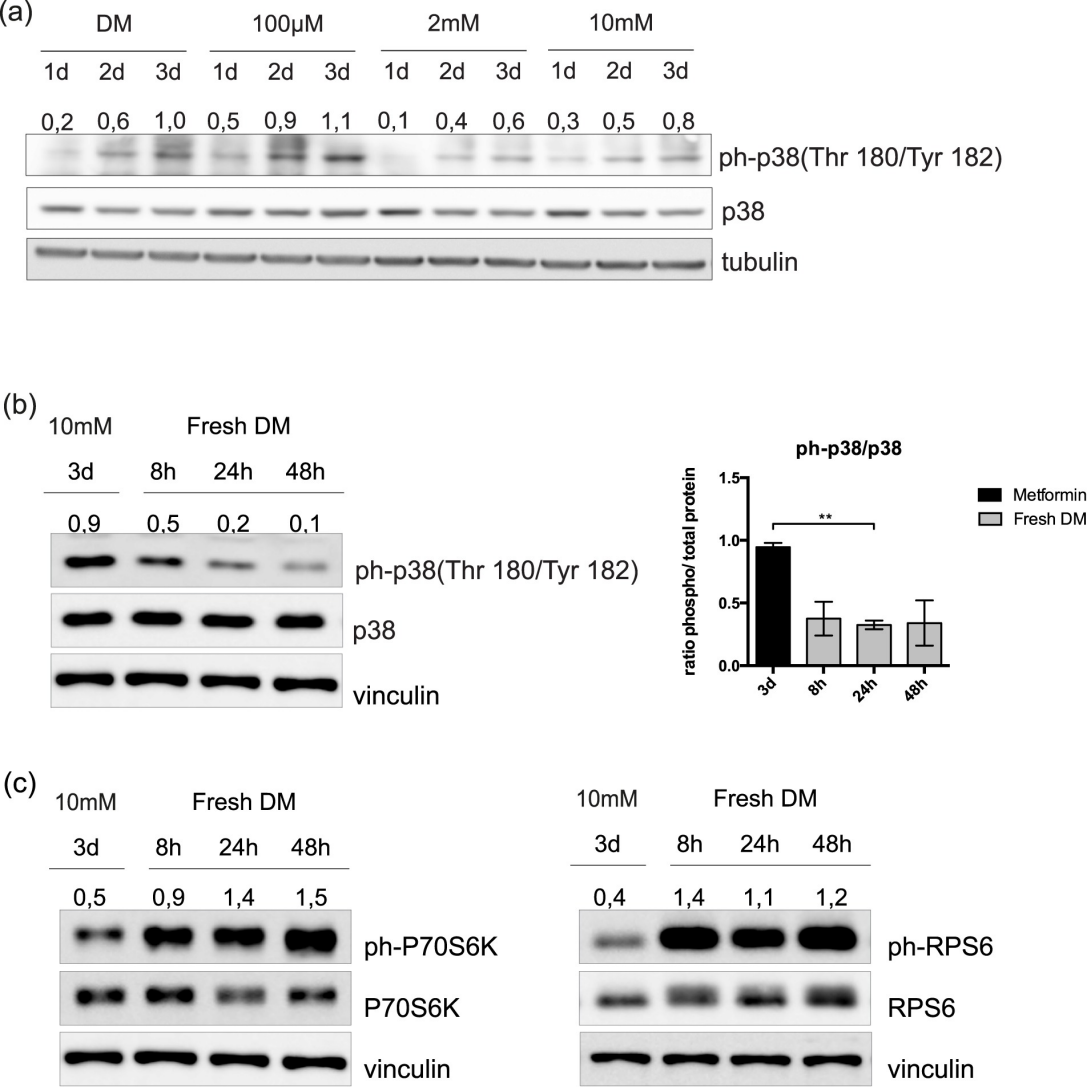
The recovery of C2C12 cells from metformin inhibition is not mediated by p38 activation. (a) C2C12 cells induced to differentiate by serum deprivation and treated in differentiation medium with 100μM, 2mM and 10mM metformin for 3 days. Metformin was refreshed every 24h and the levels of phospho-p38 and total p38 protein were analyzed in crude cell protein extracts by SDS PAGE. Tubulin was used as loading control. Numbers above each lane represent the densitometric analysis. (b) Western blot analysis and quantitation graph of phospho-p38 and total p38 protein in protein extracts from C2C12 myoblasts treated with 10mM metformin in DM for 72h and subsequently cultured for additional 8, 24 and 48 hours in fresh DM. Vinculin was used as loading control. Numbers above each lane represent the densitometric analysis (c) Western blot analysis of total P70S6K, RPS6 and their phosphorylation levels. C2C12 myoblasts were treated with 10mM metformin in DM for 72h and subsequently cultured for additional 8, 24 and 48 hours in fresh DM. Vinculin was used as loading control. Numbers above each lane represent the densitometric analysis.

To further investigate the mechanism underlying the inhibitory effect of metformin, we asked whether metformin treatment would affect the phosphorylation of p38 and, as a consequence, inhibit C2C12 proliferation and differentiation. C2C12 cells were treated for 3 days with different concentrations of metformin in DM and the phosphorylation levels of p38 were monitored by western blot. As shown in Fig 6A, the phosphorylation of p38 moderately decreases upon treatment with 2mM and 10mM of metformin, in the absence of significant alterations of the levels of p38 protein. Given that 48h after metformin withdrawal C2C12 myoblasts recover their differentiation and the expression of most myogenic markers (as shown in Fig 5B and 5C), we investigated if this recovery was caused by an increase in p38 phosphorylation. To this end, C2C12 cells were let to recover in fresh DM and monitored for the phosphorylation of p38 after 8, 24 and 48 hours. As shown in Fig 6B, the phosphorylation levels of p38 were not up-regulated in fresh DM. However, in the same extracts we observe activation of the mTOR pathway (Fig 6C). We can conclude that the reactivation and differentiation of metformin treated myoblasts is not accompanied by phosphorylation of p38.

## Discussion

Short-term calorie restriction enhances stem cell availability and activity in the muscles of young and old mice, by increasing the mitochondrial abundance and enhancing oxygen consumption [10]. This metabolic perturbation correlates with an increase in muscle satellite cells (SCs) transplant efficiency.

Ampk is a key sensor of energy status in skeletal muscle [28]. The relation between Ampk activation and myogenesis inhibition has been documented by a variety of reports. Ampk activator 5-aminoimidazole-4-carboxamide-1-β-D-ribofuranoside (AICAR) treatment of cattle myoblasts activates Ampk and suppresses myotube formation [29]. PGC1α has been reported to mediate the effect of Ampk on muscle [30,31]. Williamson et al demonstrated that the activation of Ampk by AICAR inhibited C2C12 myogenic differentiation by activating PGC1α, thus reducing nuclear Foxo3 and p21cip1 levels [31]. However, chronic treatment with AICAR favors oxidative metabolism and enhances utrophin expression in mdx mice [32]. Ampk activation is also known to cause cell cycle arrest in diverse cell types such as hepatoma HepG2 cells [33], mouse embryonic fibroblasts [34], human aortic smooth muscle cells (SMCs), rabbit aortic strips [35] and various breast cancer cell lines [19].

In skeletal muscle differentiation, cell cycle regulation and transition from the proliferation to the orderly exit from the cell cycle division are key steps which involve the regulation of many cyclins and CdKs [23]. In general, cyclins A, C, B2 and D1 as well as the kinases Cdc2 and cdk2 are down-regulated during myogenesis, while cyclin E, Cdk4 and Cdk6 do not change. Somewhat counter-intuitively, cyclin D3 expression is positively controlled by MyoD [36] and is up-regulated upon terminal differentiation, as it is required for the irreversible cell cycle arrest of differentiating myoblasts [36,37]. Gurung and colleagues have found that cyclin D3 expression induces faster differentiation kinetics and increases the levels of myogenic genes such as MyoD, Myf5, and myogenin at early stages of the differentiation process [37]. Moreover, the cell-cycle inhibitors play a fundamental role in establishing the post-mitotic state in skeletal muscle differentiation. However, p21cip1 and p27kip1 cell-cycle inhibitors appear to have diverse roles in terminal differentiation. Changes in protein levels of cell cycle regulators and Cdks reveal that the p27kip1 protein increases in non-proliferating (non-differentiating) C2C12 myoblasts as well upon differentiation, while the p21cip1 protein increases only in differentiating cells [38]. In skeletal muscle, loss of p27kip1 promotes proliferation and differentiation of satellite cells *in vitro* whereas loss of p21cip1 only affects activated satellite cells, by inhibiting their myogenic differentiation [39]. Moreover, microarray analyses of quiescent satellite cells reveal increase of p27kip1, p57kip2 and the retinoblastoma tumor suppressor protein pRB, when compared to cycling myoblasts [40].

As the activation and the asymmetric division of satellite cells are a prerequisite for the regeneration of the skeletal muscle, the ability to control the balance between muscle stem cell proliferation and differentiation would be of considerable value. Given that metformin, a calorie-restriction mimetic, favors oxidative over glycolytic metabolism via Ampk dependent and independent mechanisms [41], we asked whether the metformin induced metabolic perturbations could affect the replication and differentiation of C2C12 myoblasts. We treated C2C12 myoblasts with different doses of metformin in culture conditions that favor proliferation or differentiation and we determined their proliferation rate and differentiation potential. Our results show that myoblasts treated with metformin 2mM-10mM, have a significantly reduced growth rate without signs of apoptosis, paralleled by an inhibition of their myogenic potential. After 3 days of treatment we observe a decrease in the expression of myogenic regulatory factors MyoD, myogenin and MHC in the absence of apoptosis. In addition, formation of myotubes is impaired and the fusion index is reduced.

The inhibition of differentiation of metformin treated C2C12 myoblasts is accompanied by a reduction of p21 and cyclinD3 levels, whose up-regulation is crucial for terminal differentiation and irreversible cell cycle exit. By analyzing the distribution of cells in the different phases of the cell cycle we observed a metformin induced delay in the transition through the G2/M phase, which is accompanied by a decrease in the levels of the cyclin B and the Cdc2 p34 kinase while the expression of the cell cycle inhibitor p27, retinoblastoma-like protein p130 and Myf5 is increased. Cyclin B and Cdc2 p34 kinase are two of the most important regulators of the G2/M cell cycle transition and their levels rise during G2 phase which allows the formation of the cyclin B-Cdc2 complex and the entrance in mitosis [42]. Thus, we can speculate that metformin inhibits C2C12 proliferation by delaying the cells in the G2/M transition, an effect of metformin that has been also shown in other studies and other cell types [43–45]. Even though our results on p21 expression are consistent with the data of Williamson et al, the delayed transition through the G2/M phase observed in our experiments is not in accordance with the G1/S cell cycle block reported in Williamson work [31]. We speculate that this is a consequence of longer treatment periods (72h instead of 24h) adopted in our experimental conditions.

Previous studies have shown that increased levels of the quiescent state marker p130 and the myogenic marker Myf5 characterize the so called “reserve cells”, a sub-population of non cycling, non-differentiated myoblasts that are reversibly arrested in G0 [26]. Similarly, the levels of Myf5 are elevated in C2C12 cells whose differentiation is inhibited by leucine starvation [46]. Differently from “reserve cells”, metformin treated myoblasts, despite expressing high levels of the “reserve cell” markers p130 and Myf5, do not enter a reversible, quiescent, G0 state and keep expressing the Ki67 antigen. Moreover, we show that they are characterized by a reversible cell cycle block, as cells can recover their proliferation once metformin is removed from the medium.

In addition, C2C12 that are cultured in fresh medium after metformin treatment recover their myogenic potential and readily differentiate and form myotubes. The mitogen activated protein kinase p38 is one of the main players in myogenic differentiation and several studies have shown that p38 activation is important for myoblasts to exit the cell cycle and initiate differentiation [47–49]. In particular, it has been demonstrated that during skeletal muscle regeneration p38 recruits the SWI/SNF chromatin remodeling complex to the regulatory regions of muscle genes [50] and regulates the interaction of MyoD with E47 [51] and the phosphorylation of MEF2 [52]. In our experiments, after metformin removal C2C12 cells readily recover the activation of the mTOR pathway and differentiate without, however, any appreciable phosphorylation of p38 at the time points considered.

Hence, we propose that metformin inhibits C2C12 skeletal muscle differentiation, perturbs cell cycle progression while preventing permanent exit from the cell cycle, thus directing C2C12 cells to a state that mimics the “reserve cells” phenotype.

**Fig 7:**
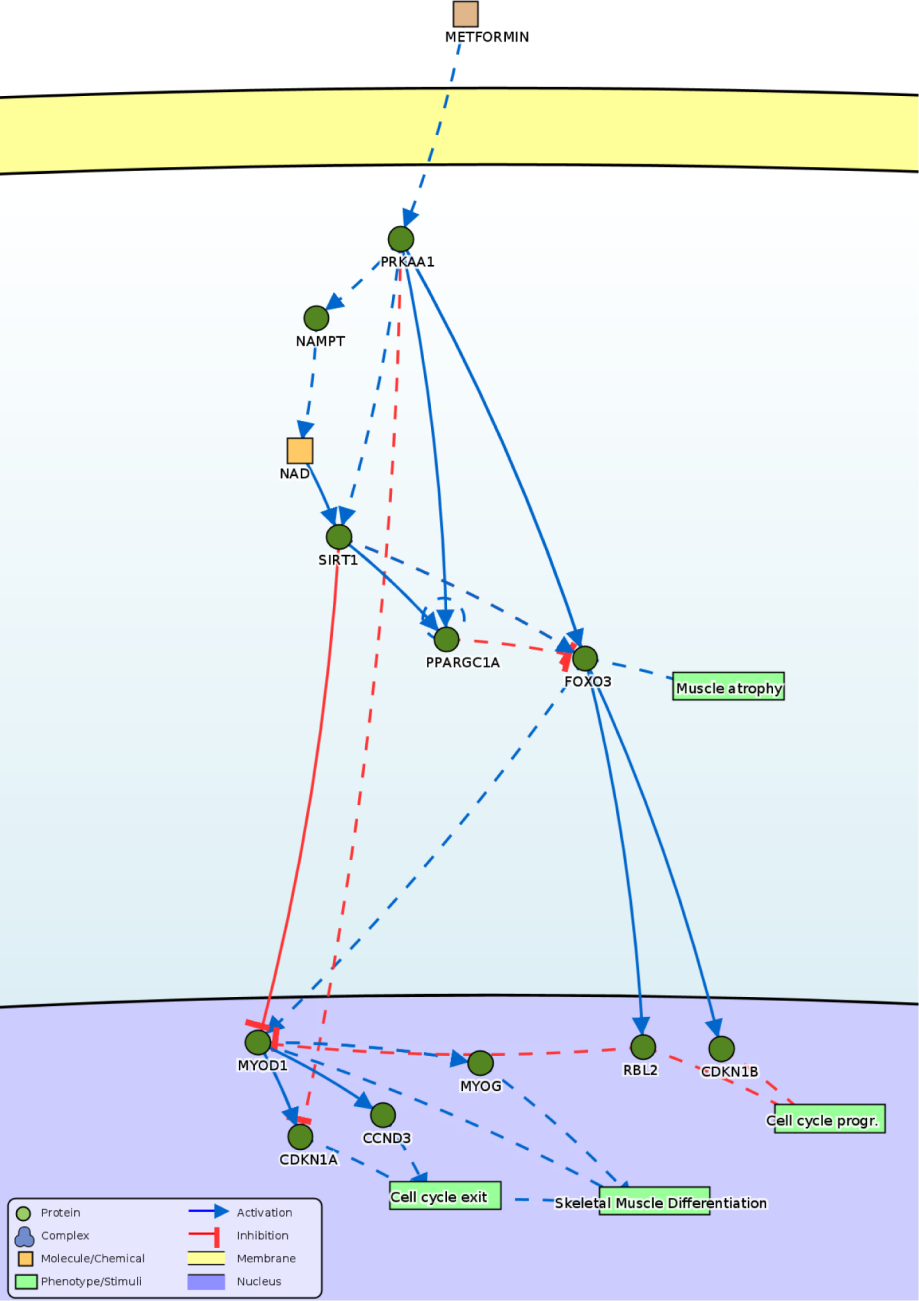
Proposed model consistent with the presented data. The model represents the causal relationships that can explain the experimental observations obtained by treating the cells with high doses of metformin (5 and 10mM), as extracted from the SIGNOR database, available at http://signor.uniroma2.it [54]. Activations and inhibitions are depicted as blue arrowhead and red hammerhead, respectively. Direct relationships are represented by continuous lines while indirect ones by dashed lines. Proteins are labeled with their UniprotKB gene names. The correspondence with the common name used in the text is the following: PRKAA1 = Ampk, NAMPT = Nampt, SIRT1 = Sirt1, PPARGC1A = PGC1a, FOXO3 = Foxo3, MYOD1 = MyoD, MYOG = myogenin, CCND3 = cyclin D3, CDKN1A = p21, RBL2 = p130, CDKN1B = p27.

Our results are consistent with reports that connect the activation of AMP-activated kinase (Ampk) to impaired skeletal muscle differentiation. In particular, Fulco et al have demonstrated that glucose restriction inhibits myoblasts differentiation through the Ampk/Nampt/Sirt1 axis [53]. Moreover, our work highlights that metformin induces a reversible block of the cell cycle that is incompatible with terminal differentiation. Combining our observations with those of Fulco et al [53] and Williamson et al [31] we propose the model in Fig 7 illustrating the causal relationships that link the activation of Ampk to cell cycle perturbation and inhibition of differentiation. The observations by Cerletti et al [10] that calorie restriction (CR) increases the number of isolated SCs, enhances their myogenic function *ex vivo* and transplant efficiency cannot be fully explained by this model. In those experiments both in young and aged CR-treated mice, the enhanced myogenic function is accompanied by an increased fraction of satellite cells expressing the conserved longevity/metabolic regulators Sirt1 and Foxo3. The differences in the experimental systems and in the type of perturbations may explain the different results. However, altogether these reports stress the important impact of energy regulation and of its sensor Ampk on myoblast differentiation.

## Supporting information

S1 Fig Trypan blue assay of C2C12 cells treated with different doses of metformin. (TIF)
