# Supplementary figures and images for: Regulation of myoblast differentiation by metabolic perturbations induced by metformin
Source: PLoS One. 2017 Aug 31;12(8):e0182475. doi: 10.1371/journal.pone.0182475 (PMC5578649; doi:10.1371/journal.pone.0182475)

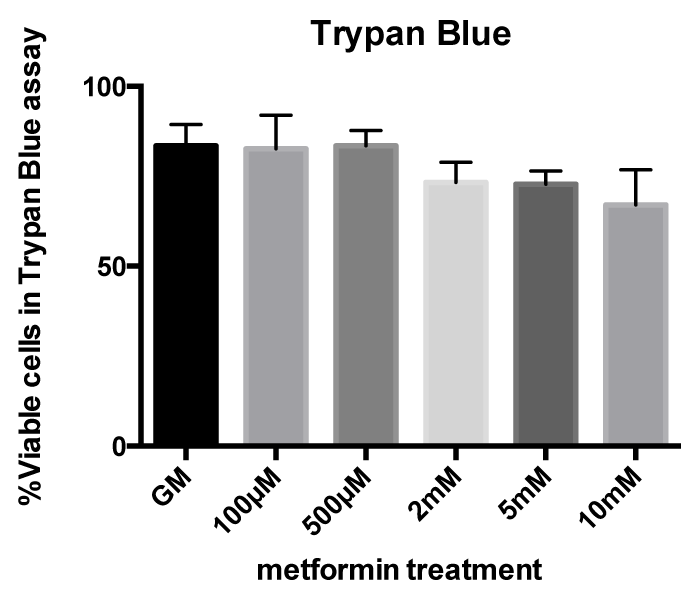

Supplement: S1 Fig — (TIF) [file pone.0182475.s001.tif]
